# Prolapsus Ani Treated after the Manner of Mr. Hey

**Published:** 1830-01-01

**Authors:** 


PROLAPSUS ANI TREATED AFTER THE MANNER OF MR. HEY. By Dr. MACFARLANE.

In the last number of our respected contemporary of Glasgow, is an interesting case of prolapsus ani, occurring in one of the pauper patients of that town.

*Case.* W. A. shoe-maker, æt. 54, became a district patient in the beginning of February. The gut descended for more than two inches on every attempt to evacuate the bowels, accompanied with considerable pain and tenesmus. When he remained for a few minutes in an erect position, the same displacement took place slowly, although no propulsive efforts were employed; this, however, could be prevented by pressure. The first thing projected from the anus was a circular fold of the mucous membrane of the rectum, at its verge, of a livid colour and tuberculated appearance, and this was soon followed by the complete descent of the bowel and hæmorrhage from innumerable points. The recumbent posture and gentle but continued pressure for a few minutes generally effected the reduction of the prolapsus, although at an earlier period it often continued irreducible for hours. His general health was much impaired, and the constant irritation and almost daily attacks of hæmorrhage, disabled him from following his employment.

"On examining the anus after the gut was replaced, the surrounding integuments were found extremely relaxed. There existed such an unnatural looseness in the attachment of the skin around the anus, to its corresponding cellular membrane, that it could be easily drawn out with the fingers in the form of one or more large flaps. Having succeeded in two similar cases, which came under my care in the Royal Infirmary, during the summer of 1826, in completely curing the disease, by cutting off the loose integuments, as recommended by the late Mr. Hey,* I determined to try it in this case. The skin was drawn as far out as possible into broad flaps, and cut off with the scissors in a circular direction, until all the superfluous integument was removed, including a portion of the livid and tuberculated fold of mucous membrane which was projected from within the sphincter. The pain was trifling, and only a few drops of blood were lost. A soft compress and T bandage were applied, and he was strictly confined to bed. For a few days, a partial procidentia took place on every attempt to go to stool. He had a good deal of pain and inflammation around the anus, attended with complete retention of urine, which required the frequent introduction of the catheter. In ten days after the operation, he was able to walk about, and void his stools, without any return of the disease, and in three weeks he was perfectly cured. Pressure was continued to the part for some time longer—occasional doses of castor oil were prescribed, and he was enjoined to avoid straining at stool.

"There will generally be found in obstinate and long-continued forms of this disease, a great relaxation in the connexion of the rectum at its lower part, with the surrounding textures. This circumstance, although it may not be the original cause, is sufficient, in many cases, to account for the continuance of the displacement in chronic and inveterate cases, although I believe it is generally accompanied by a diminished power of the sphincter. If the

* "Practical Observations in Surgery, 2d edit. p. 444."

rectum, in consequence of being much irritated, as in various bowel complaints, ultimately becomes relaxed, the tenesmus, which is an invariable attendant, may so overcome the sphincter, as to give rise to a procidentia. But when, as in the case now detailed, the erect position is sufficient to cause a descent of the gut, we have grounds for believing, that besides the relaxed state of the rectum, there exists a want of power in the sphincter muscle, which part, along with the levator ani, is mainly instrumental in maintaining the rectum in its natural situation. In the cases detailed by Mr. Hey, there existed in combination with relaxation of the integuments, one or more livid tubercles at the verge of the anus, which were also removed. He expected from this operation, that inflammation of the surrounding cellular texture would be excited, the attachments of the rectum consolidated, and the power of the sphincter improved. In a majority of cases, the disease will be found to yield (although the cure is often tedious and protracted) to the local applications and internal remedies usually employed. Should it continue, however, as sometimes happens after the exciting cause has been removed; we will occasionally find that the loose state of the skin around the anus, and the relaxed attachments of the rectum, at its termination, become the primary causes of the continuance of the disease. It is, I conceive, in such circumstances that this simple operation may be beneficially adopted."

The case and remarks appended are practically interesting.

